# Even Low Amounts of Amorphous Lignocellulose Affect Some Upper Gut Parameters, but They Do Not Modify Ileal Microbiota in Young Broiler Chickens

**DOI:** 10.3390/ani15060851

**Published:** 2025-03-16

**Authors:** Valéria Farkas, András Mayer, Judit Poór, Eszter Péterné Farkas, Kesete Goitom Tewelde, Brigitta Kiss, Nikoletta Such, László Pál, Gábor Csitári, Károly Dublecz

**Affiliations:** 1 Institute of Physiology and Nutrition, Department of Animal Nutrition and Nutritional Physiology, Georgikon Campus, Hungarian University of Agriculture and Life Sciences, Deák Ferenc Street 16, 8360 Keszthely, Hungary; farkas.valeria.dr@gmail.com (V.F.); peterne.farkas.eszter@uni-mate.hu (E.P.F.); kesetehac@gmail.com (K.G.T.); brigitta.kiss@ubm.hu (B.K.); such.nikoletta.amanda@uni-mate.hu (N.S.); pal.laszlo@uni-mate.hu (L.P.); csitari.gabor@uni-mate.hu (G.C.); 2 Institute of Animal Science, Hungarian University of Agriculture and Life Sciences, Guba Sándor Street 40, 7400 Kaposvár, Hungary; andras.mayer@jrs.co.at; 3 Institute of Mathematics and Basic Science, Georgikon Campus, Hungarian University of Agriculture and Life Sciences, Deák Ferenc Street 16, 8360 Keszthely, Hungary; poor.judit@uni-mate.hu

**Keywords:** amorphous fiber, broiler chicken, production traits, intestine function, microbiota

## Abstract

A feeding trial was carried out using 576 Ross 308 male chickens to test the effects of an amorphous lignocellulose (LC) product (Arbocel). Three dietary treatments (control, 0.5% LC (LC 0.5), and 0.8% LC (LC 0.8) were used, and the diets were fed in eight replicate pens of 24 chickens. The LC treatments did not modify the weight gain of the chickens, but they tended to decrease the feed intake and FCR in the grower phase. The high swelling characteristics of LC resulted in lower dry matter (DM) contents in the crop *(p* < 0.002) and gizzard (*p* < 0.047) on day 14 and day 24, respectively. The lower pH in the gizzard and duodenum of LC diets suggests longer retention time and more HCl secretion in the proventriculus and gizzard. Except for α-amylase on day 24, the treatments did not modify the digestive enzyme content of the jejunum. The microbiota diversity and microbiota composition of ileal contents were affected only by the age of birds. The results suggest that the LC product can exert its potential positive effects mainly in the upper part of the digestive tract due to its special physical characteristics. LC can slow down the passage rate in the gizzard and modify the digestion and the dynamics of water absorption from the jejunum and ileum.

## Simple Summary

Many research results have proved that stimulating the gizzard by structural fibers, course particles, or whole seeds has several positive results on digestion and gut health. The soluble fiber fractions of poultry feed increase gut viscosity, decrease digestion, modify the gut microbiota, and impair litter quality. A special group of fiber sources and additives are processed lignocelluloses with a high swelling potential. These products are used in the practice at low inclusion rates, but their effects are not fully understood yet. In this trial, the lignocellulose (LC) product Arbocel was fed at 0.5 and 0.8% to broiler chickens, and besides the production traits, several gut parameters were evaluated, focusing mainly on the upper digestive-tract parts. Despite the low inclusion rates, feeding LC tended to significantly modify the dry matter of the crop, gizzard, jejunum, and ileum digesta, as well as the digesta pH in the gizzard and in some small intestine parts, and it increased the α-amylase activity of the jejunum. However, the bacteriota of the ileum was not affected by the LC treatments. It was concluded that Arbocel can improve the digestion of chickens by slowing down the passage rate, which can improve the digestion and absorption of nutrients.

## 1. Introduction

The term “dietary fiber” means non-digestible polysaccharides, such as resistant starch, lignin, soluble, and insoluble non-starch polysaccharides (NSPs). In poultry species, dietary fiber may have both beneficial and harmful effects on the digestion process, depending on the characteristics of the fiber [1]. It is well known that the feeding of structural fiber could affect gizzard function, digestive tract development, enzyme secretion, and gut pH. Fiber is also essential in stimulating gut motility, and the peristaltic and antiperistaltic movements of the digesta [2]. On the other hand, high fiber could be depressive and lower the efficiency of the gastrointestinal enzymes to reach their substrates [3,4]. Soluble fibers in viscous cereal grains or industrial by-products increase the viscosity of the gut content, decrease the rate of diffusion of digestive enzymes, and decrease interaction of the digested molecules with the mucosal surface [5]. This negative effect can be decreased if exogenous NSP-degrading enzymes are used [6].

According to research, functional fiber refers to isolated, extracted, or synthetic fiber that can provide health benefits, along with several physiological functions [7,8]. Lignocellulose-based crude fiber concentrates are prepared with a unique milling technique, which results in a high water-binding capacity for the products [9]. The lignocellulose content of plant cell walls is mainly composed of the insoluble cellulose, the partly water-soluble hemicellulose, and the biopolymer lignin [10]. It is the most abundant renewable carbon source on earth and a potential resource for the sustainable production of chemicals and fuels [11]. In addition, it is also possible to use lignocellulose as a dietary feed additive for humans and for animals, with potentially positive effects on their digestive physiology. Due to the quick passage rate, the short digestive tract, and the anatomical structure of the caeca, chickens have only limited ability to utilize insoluble NSP and limited microbial cellulolytic activity in the hindgut [4,12,13,14,15].

It has been shown that dietary lignocellulose at a low inclusion level might stimulate the development of the digestive tract, the passage rate, and enzyme activities in pullets and laying hens [16], and it might enhance mucosal development in broilers [17,18]. Some studies reported that dietary lignocellulose can beneficially affect the litter quality by lowering the excreta moisture content [18,19,20]. Inclusion of lignocellulose in chickens’ diet at 0.5% promoted the growth of *Lactobacillus* spp. and *Bifidobacterium* spp., and it reduced the number of *E. coli* and *Clostridium* spp. in the caeca. It also increased the concentration of short-chain fatty acids (SCFAs) and lactic acid content of the ileal and cecal digesta [18,19,21]. Feeding energy- and a nutrient-reduced diets containing 10% lignocellulose reduced body fat content and improved the laying performance in dual-purpose laying hens [22,23].

In several European countries, amorphous lignocellulose-based feed additives are used in broiler diets. However, the exact mechanism behind the positive effects is not known. Therefore, the goal of the current study was to investigate the effects of feeding lignocellulose in the practical incorporation range (0.5 and 0.8%) on the production traits, development of gastrointestinal organs, digestive enzyme activity of the jejunum, and ileal microbiota composition. Since amorphous lignocellulose is believed to be advantageous mainly in young animals, the evaluation of gut parameters was carried out at the end of the starter and grower phases.

## 2. Materials and Methods

### 2.1. Experimental Design and Dietary Treatments

A total of 576 male broiler chickens (Ross 308) were used in a feeding experiment. The day-old chickens were obtained from a commercial hatchery (Gallus Ltd., Devecser, Hungary) and were transported to the experimental farm of the Institute of Physiology and Nutrition, Hungarian University of Agriculture and Life Sciences (Georgikon Campus, Keszthely, Hungary). The birds were randomly allocated to 24 floor pens in a windowless building. The size of the pens was 2 m^2^, and the 24 chickens per pen resulted in 12 birds per m^2^ stocking density. Chopped straw was used as bedding material.

In the study, 3 dietary treatments with 8 replicate pens were used. Besides a maize–soybean-based control diet (**C**), an amorphous lignocellulose (LC) product (Arbocel^®^, Rettenmaier & Söhne GmbH + Co. KG, Rosenberg, Germany) was included at a level of 0.5% (LC 0.5) and 0.8% (LC 0.8). The fiber content of the product is 70%, containing more than 20% lignin.

The starter diets (0–11 days) were fed in mash; the grower (12–24 days) and finisher feeds (25–42 days) were in pelleted form. Feed and water were available ad libitum throughout the experiment. Diets were formulated according to the nutritional recommendations of the breeder company [24]. The composition and measured nutrient content of the experimental diets are shown in Table 1. The LC was supplemented into the diets at the expense of corn, but the low inclusion rates did not modify the nutrient content of the diets (Table 1) The climatic parameters were maintained according to the breeder’s recommendations [24]. The room temperature was 34 °C on day 0 and was reduced gradually to 24 °C on day 18. In the first week, the light intensity was 30 lux, and it was 10 lux afterward. The light period was 23 h/day in the first 7 days and 20 h in the remaining period. The trial was approved by the Animal Welfare Committee of Georgikon Campus, Hungarian University of Agriculture and Life Sciences, under the license number MÁB—2/2020.

### 2.2. Measurements and Samplings

The live weight (BW) of chickens was measured at the start and at the end of each phase, using an electronic poultry scale (VEIT BAT1, VEIT Electronics, Moravany, Czech Republik). Feed intake (FI), body weight gain (BWG), and feed-conversion ratio (FCR) were measured on a pen basis for each phase and for the entire experimental period. Commercial scales were used for the measurements of excreta, remnant feed, and the carcass composition. The accuracy of the live weigh and feed measurement was 1 g, while that of the excreta and carcass part was 0.1 g. FI was calculated as the difference between the provided and remnant feed, BWG as the difference between the final and initial BW of animals, and FCR was the quotient of FI and BWG. The mortality was recorded daily. The European Production Efficiency Factor (EPEF) was calculated using the following formula:

(1) $$EPEF=\frac{liveability\;(\%)\times final\;body\;weight\;(kg)}{FCR(kg/kg)\times length\;of\;fattening\;(days)}\times 100$$

On days 14 and 24, representative excreta samples were collected from each pen. To obtain clear samples without bedding material, the litter of pens was covered with nylon foils for 5 h. Around 150–200 g excreta were collected from the different parts of the pen. The collected samples were homogenized and 50 ± 1 g frozen at −20 °C. From these samples, the dry matter content of the excreta was determined in a drying oven (24 h, 105 °C). On days 14 and 24, two chickens per pen with similar body weights were selected randomly and killed by CO_2_ asphyxiation. The abdominal cavity was opened immediately, and the digesta contents of the different gut segments were collected. The digesta contents were weighed in fresh form and also after drying. The empty weight of crop, gizzard, duodenum, jejunum, and ileum was also weighed. The duodenum was defined as the part of the small intestine between the gizzard and bile duct junction, the jejunum till the Meckel’s diverticulum, and the ileum between the Meckel’s diverticulum till the ileocecal junction.

The pH of each digestive part contents (crop, gizzard, duodenum, jejunum, and ileum) was determined from the fresh intestinal contents. The fresh intestinal contents were homogenized, diluted with distilled water (1:5), and shaken by hand for 1 min. The pH was measured with a SNEX electrode (pH200A), a portable pH meter equipped with a CS1068 SNEX pH sensor (CLEAN Instruments, Shanghai, China).

The α-amylase, lipase, and trypsin activities of the jejunal digesta were measured. The activity of α-amylase (EC 3.2.1.1; Sigma-Aldrich; St. Louis, MO, USA) was determined using the colorimetric method, as described by [25]. One α-amylase activity unit was defined as the amount of amylase that liberates 1.0 mg of maltose from starch in 3 min at pH 6.9 at 20 °C. Lipase activity (EC 3.1.1.3; Sigma-Aldrich; St. Louis, MO, USA) was measured via the titrimetric method [26]). One-unit lipase activity was defined, as it hydrolyzes 1.0 micro equivalent of fatty acids from a triglyceride in one hour at pH 7.7 at 37 °C. Trypsin activity (EC 3.4.21.4; Sigma-Aldrich; St. Louis, MO, USA) was measured by a spectrophotometric method [27]. The trypsin activity unit was measured with a N-α-Benzoyl-L-Arginine Ethyl Ester (BAEE) solution. One trypsin unit is defined as the amount of enzyme which increases the absorbance (wavelength: 253 nm) by 0.001 per minute at pH 7.6 and 25 °C. Protein concentration of the intestinal content was determined by the modified Biuret–Lowry method described by Ohnisi and Barr [28] and expressed as mg protein per g of wet digesta content.

Ileal chymus contents were collected for analysis of microbiota composition. The samples were taken from the middle part of ileum, homogenized, and stored in sterile containers at −80 °C. Before DNA extraction, the samples of two birds of the same pen were pooled, resulting in 8 replicates per treatment.

### 2.3. DNA Extraction and Bacterial 16S rRNA Sequencing

The AquaGenomic Kit (MoBiTec GmbH, Göttingen, Germany) was used for DNA extraction and purified using KAPA Pure Beads (Roche, Basel, Switzerland) according to the manufacturer’s protocols. The DNA concentration of samples was determined by Fluorometric quantitation (Qubit 3.0 Fluorometer with the Qubit dsDNA HS Assay Kit—Thermo Fisher Scientific Inc., Waltham, MA, USA). To amplify the variable region V3–V4 of the 16S rRNA gene, 341F and 785R primer pairs were used [29]. Amplicons were quantified and qualified by using the High Sensitivity D1000 ScreenTape on the TapeStation 2200 instrument (Agilent Technologies, Santa Clara, CA, USA). Sequencing was performed on the Illumina MiSeq platform (MiSeq Reagent Kit V.3, 600 cycles; Illumina Inc., San Diego, CA, USA) according to the manufacturer’s recommendation.

### 2.4. Bioinformatics Analysis

The QIIME2 Pipeline (Quantitative Insights into Microbial Ecology—Version 2020.2.) was used to analyze the raw reads [30,31]. Quality filtering of raw sequences occurred using the q2—demux plugin, followed by denoising with Deblur with the QIIME2 default setting [32]. High-quality sequences were clustered into operational taxonomic units (OTUs). Representative sequences were found using the Deblur denoise—16S method. VSEARCH algorithm was used for open-reference clustering, based on a 97% similarity to the SILVA (release 132) reference database [33].

Diversity indices (alpha and beta) were calculated using QIIME2 version 2020.2 software package and MicrobiomeAnalyst (https://www.microbiomeanalyst.ca/, accessed on 1 September 2020) software [34]. For beta diversity, the variations in the microbial composition among groups were investigated using the Bray–Curtis dissimilarity method, and this distance was presented using principal coordinate analysis (PCoA). The significance of the fractions in the PCoA plot was tested using permutational multivariate analysis of variance (PERMANOVA).

### 2.5. Statistical Analysis

The production parameters were evaluated with one-way analysis of variance (ANOVA) and Tukey’s post hoc tests, conducted by using IBM SPSS 29.0 (IBM Corp. Released, Armonk, New York, 2024). Significance was set at *p* < 0.05, whereas a *p*-value between 0.05 and 0.10 was considered a trend. Mortality results were assessed using independent-samples proportion test at the level of *p* = 0.05. The microbiota alpha diversity indices of the ileum chymus were compared using two-way ANOVA test with Tukey’s HSD multiple group comparison’s post hoc test, using the dietary treatments (C, LC 0.5, and LC 0.8) and the age of birds (day 14 and day 24) as main factors. All data were tested for normality and homogeneity of variances by Shapiro–Wilk and Levene’s test. Two-way analysis of variance was performed to test the taxa-abundance differences and to explore the differences between the communities derived from the different ages and different treatments. For all taxa, the Benjamini–Hochberg (BH) procedure was used to keep the false discovery rate (FDR) at FDR < 1%.

## 3. Results

### 3.1. Production Traits

The average weight of day-old chickens was 48.7 g, without significant differences between the treatment groups. The LC supplementation failed to cause significant differences in body weight, body weight gain, feed intake, and the feed-conversion ratio (FCR) of animals (Table 2). However, in the grower period, the FI of the L C 0.5 and LC 0.8 groups and the FCR of the LC 0.5 treatment were tendentially lower (*p* ≤ 0.1). The mortality values were low in all treatment groups, and the two sample proportion tests revealed that there was no significant difference (C-LC 0.5: *p* = 0.252; C-LC 0.8: *p* = 0.297; LC 0.5-LC 0.8: *p* = 0.736) in the death rate of birds. The EPEF was 436, 455, and 435 in the treatment groups C, LC 0.5, and LC 0.8, respectively.

### 3.2. Weight, Digesta Dry Matter, and Digesta Content of the Different Gut Segments

The weight of the gut segments was not affected. As a tendency, the duodenum weight increased on day 14 in both LC-supplemented groups (*p* = 0.085) (Table 3). The LC treatments numerically increased the weight of the gizzard on days 14 and 24, but the differences were not significant. On day 14, treatment LC 0.8 showed a tendency to decrease the DM content of the crop significantly and that of the gizzard compared with the control group. The opposite trend was found for the DM of jejunum. In this gut segment, feeding LC at 0.5% resulted in significantly higher DM. The tendency for the digesta DM to increase the effect of LC also remained in the ileum (Table 3). On day 24, the LC 0.8 treatment decreased the DM of the gizzard content, but opposite to the day 14 results, the DM of the jejunal and ileal contents decreased on day 24 if LC was fed (Table 4). No significant differences were observed in the weights of gut contents (Table 3 and Table 4).

The dry matter content of excreta was numerically lower in the LC treatments compared with the control group, but the differences were not significant. The DM contents on day 24 were similar (Table 5).

### 3.3. Digesta pH and Enzyme Activity of the Jejunal Content

The pH of the crop content of chicks was not influenced by the dietary treatments (Table 6). On the other hand, in the gizzard and in the ileum, on day 14, the pH was significantly lower in the LC 0.5 dietary group compared to the C and LC 0.8 groups. On day 24, LC supplementation caused in the duodenum significantly lower (LC 0.5 and LC 0.8) and in the jejunum significantly higher (LC 0.8) pH. The results of the gizzard on day 14 and the duodenum on day 24 suggest longer digestion time in the gizzard and, in this way, more hydrochloric acid secretion.

The trypsin activity in the jejunum was not affected by the treatments; however, numerically, it was lower in the LC 0.5 group compared to the C and LC 0.8 (Table 7). The α-amylase and lipase activity in both sampling time points was numerically higher in the LC 0.5 dietary group compared to the C and LC 0.8 groups, but the difference was significant only on day 24 (Table 7).

### 3.4. Microbial Diversity of Ileum

**Figure 1:**
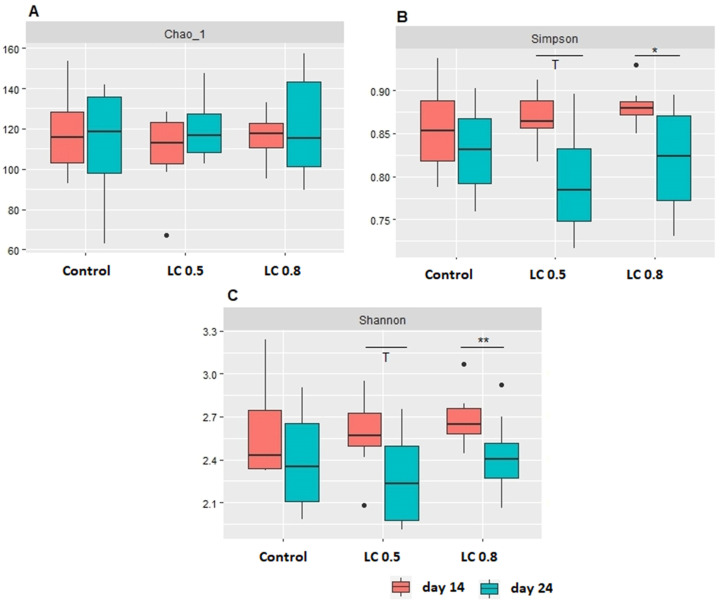
Alpha diversity, including Chao (**A**), Simpson (**B**), and Shannon (**C**) indices, of the ileal microbiota of broiler chickens, as affected by the dietary treatments (C—control; LC 0.5—0.5%; LC 0.8—0.8% lignocellulose supplement) and age (red, day 14; blue, day 24) of birds. Statistical significance was determined by two-way ANOVA and the Tukey multiple-comparisons test. The different superscript notations on the boxplot represent a significant difference (* *p* < 0.05; ** *p* < 0.01; 0.05 < *p* < 0.10 were considered a trend (T)).

The alpha-diversity indices (Chao_1, Simpson, and Shannon) indicated that only the age of chickens had a significant effect on the bacterial diversity (Figure 1A–C).

**Figure 2:**
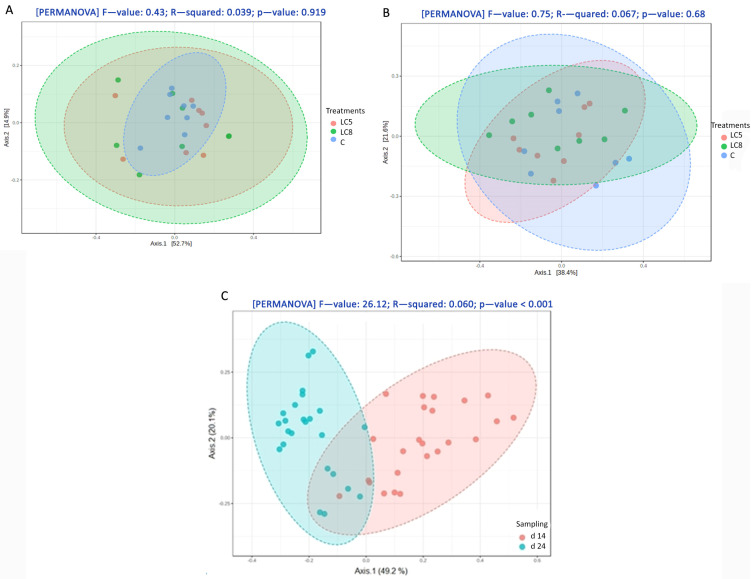
Principal coordinate analysis (PCoA) based on Bray–Curtis dissimilarity matrix in the ileal chymus, grouping by dietary treatment on day 24 (**A**) and grouping by dietary treatment on day 14 (**B**). Permutational multivariate analysis of variance (PERMANOVA) was used to analyze spatial variation in beta diversity of the samples. PCoA of the broiler’s gut microbial community composition of different ages (**C**), based on the Bray–Curtis distances, showed distinct clusters (red, day 14; blue, day 24).

Beta-diversity based on principal coordinate analysis (PCoA) ordination using the Bray–Curtis dissimilarity matrix also showed significant differences (PERMANOVA global R = 0.60, *p* = 0.001) between the two age categories (Figure 2C). Dietary treatments failed to cause significantly different bacterial community structures in ileum (Figure 2A,B).

### 3.5. Ileal Microbial Abundances

In the ileal content, *Firmicutes* was the dominant phylum, accounting for about 99% of the total detected phyla (Table 8). The dietary treatments did not cause significant differences. The only significant difference at the phylum level was the increase in *Verrucomicrobia* abundance on day 24, compared with the result of day 14.

At the genus level, *Lactobacillus* was dominant, followed by *Staphylococcus*, *Enterococcus*, *Streptococcus*, *Corynebacterium_1*, *Candidatus_Arthromitus*, and *Weissella* (Table 9). The bacterial composition was affected only by the age of the animals. Among genera above 1% abundance, Lactobacillus increased, while the abundance of *Staphylococcus*, *Enterococcus*, and *Candidatus_Arthromitus* decreased on day 24, compared to day 14.

## 4. Discussion

The fiber evaluation system of poultry diets is not accurate, based mainly on crude fiber (CF) content of the feedstuffs. No exact CF requirements and limits exist for birds of different species and age categories. CF is underestimating the real fiber content of feedstuffs, which is a source of inaccuracy during diet formulation. Developing a more accurate fiber evaluation system is difficult, since dietary fiber is very complex, containing soluble and insoluble fractions; crystalline cellulose with different lignification; and a changing ratio of amorphous matrix of hemicellulose, xylans, glucans, and pectins [35]. Dietary fiber can modify the feed intake, gizzard function, digestive enzyme secretion, nutrient digestibility, gut health, and several gut parameters (morphology, viscosity, gut associated immune system, etc.). The effect of the different fiber sources depends on the inclusion rates and several chemical and physical characteristics (soluble, insoluble, structural, particle size, and lignification) [36].

The fiber-degrading capacity of birds is limited due to the high feed passage rate and the short colon [4,37]. The main place of bacterial fermentation is the caeca, but unlike mammals, not all the non-digested nutrients can get into the caeca, only the very fine and soluble fractions [13].

Insoluble fibers are assumed not to be degraded in the chicken’s digestive tract and are used as diluents [14]. However, specific lignocelluloses (Arbocel and OptiCel), as feed additives, are used in broiler-chicken, laying-hen, and turkey diets at inclusion rates below 1%, as such rates seem to have positive effects on the production traits, gut bacteria, mucosa development, and litter quality [17,18,21]. This special LC originates from woods, after a specific processing procedure that results in more than 60% fiber content and high swelling and water-binding capacity. The theory behind the positive effects is that these LC products fill up the gizzard; modify the feed intake, the HCl, and the pepsin secretion of the proventriculus–gizzard, slowing down the passage rate; loosen the digesta; and improve the penetration of digestive enzymes to the substrates [4]. It can improve digestibility, and, in this way, it can also support the competitional exclusion of pathogenic bacteria in the small intestine, mainly in the ileum. Since the effects of amorphous LCs are located mainly in the upper digestive-tract parts in our study, the evaluation was focused on these gut segments. The other new aspect of our trial was that the response of young chickens was measured in two age categories. In our case, LC was not used as a diluent, and at 0.5 and 0.8%, which corresponds to the practical inclusion rates. The experimental diets were isocaloric and isonitrogenous.

### 4.1. Production Traits

The weight gain of chickens was not affected by the LC treatments in this trial, but in the grower phase, the animals had a tendency to consume less feed from the LC-supplemented diets. The FCR was at the LC 0.5 treatment the best. It seems that chickens in the grower phase were more sensitive to the product. The reason for the feed intake reduction could be the swelling property of LC and the quicker filling of the gizzard. It is well known that the gizzard plays an important role in birds as a feed-intake regulator [38]. In spite of the lower FI of LC 0.5 birds in the grower phase, the growth rate of this group was numerically the highest. It was the reason for the lowest FCR of the LC 0.5 group. Bogusławska-Tryk et al. [21] used 0.25, 0.5, and 1% inclusion rates of Arbocel in the broiler finisher diets (day 21 to day 42), and similarly to our findings, the 0.5% inclusion rate gave the best results regarding the bacteriota composition, SCFA, and lactic acid content of the ileum and caeca. However, in that trial, no effects on the production traits were observed. Röhe at el. [23] used higher inclusion rates of LC (0.8, 5, and 10%) in a trial with 10-week-old slow-growing broiler chickens. Surprisingly, no effects were found in the production traits, despite the fact that 10% LC decreased the digestibility of dietary protein, DM, and gross energy. In contrast with the results of Bogusławska-Tryk et al. [21], the inclusion rate of LC showed a significant negative correlation with the CSFA production in the caeca. In the experiment of Makivic et al. [18], 0.4 and 0.6% inclusion of Arbocel in the starter (day 0 to day 13) and grower (day 14 to day 28) diets of Cobb 500 broiler chickens resulted in significant positive effects in the growth and FCR. However, the crude protein, amino acid, and energy content of diets were not identical in that trial, so the results were not exclusively related only to the LC treatments. Soltan et al. [39] did not find LC effects on the production parameters in a 6-week-long feeding trial, at 0.5 and 1% LC levels. The reason for the contradicting results is partly that most of the previous experiments evaluated the LC effects for the whole production period, and the design (dilution or isonitrogenous, isocaloric diets) and the animals (commercial broilers vs. slow growing chickens) were different.

### 4.2. Gut Characteristics

Feeding LC did not result in changes in the weight of the gut segments on day 14 and 24. Only the duodenum weight increased tendentially on day 14 in the LC-supplemented group, thus suggesting a longer transit time. The presence of 0.5% and 0.8 LC in the diets on day 14 and 24 resulted in increased gizzard weight in comparison to the controls, but the differences were not significant. These results correspond to the recent studies indicating that stimulation of the gizzard by insoluble fiber is related closely to its physical structure. Dietary fiber affects the length and weight of the gastrointestinal tract (GIT) [40]. There is also strong evidence that the differences in the weight of organs are highly related to differences in the type of fiber [4]. Diets rich in fiber content may produce greater dilatation of proventriculus with the increase in size and its contents [41]. The coarse fiber particles are selectively retained in the gizzard, which ensures a complete grinding and a well-regulated feed flow and secretion of digestive juices [1]. Röhe et al. [22] supplemented the diets of dual-purpose hens with 10% LC, which resulted in a significant increase in the relative weight of the gizzard, small intestine, and large intestine. However, these results are not comparable with ours, due to the different ages of the chickens and inclusion rates of LC. The LC treatments in this experiment resulted in only a slight increase in fiber content at diet level, and therefore it is difficult to measure significant differences in the gut parameters. However, our results with young broiler chickens support the theory that LC, even at low inclusion rate, can stimulate the gizzard and, on day 14, even the function of the duodenum.

On day 14, the DM content of crop content decreased significantly, and that of the jejunum increased if LC was in the feed. The more water in the crop content was the result of the high water-binding and swelling characteristics of LC, but there is no explanation for the DM change in the jejunum. On day 24, LC significantly increased the water content of the gizzard and jejunum, and it also showed a tendency to do so in the ileum contents. These results are in line with the expectations, but they are opposite to the jejunum results on day 14. The results suggest that LC can modify not only the transit time of the feed but also the dynamics of water absorption from the jejunum and ileum. No such results on amorphous LC at these age categories have been found in the literature. In opposition to some published data [18,22], LC treatments had no effect on the dry matter content of excreta on day 14 or day 24. The results, however, are in line with those of Röhe et al. [23]. The reason for the contradicting results is the differences in the animals (broiler chickens and laying hens), the age of the chickens, the composition of basal diets, and the origin of the samples (excreta vs. manure). The digesta content of the crop tended to decrease with the LC 0.8 treatment compared with the two other treatments. No other changes were observed, which means amorphous celluloses have only a limited effect on the amount of the dried intestinal content; rather, they modify the water content and the physical characteristics of the digesta.

### 4.3. Digesta pH and Enzyme Activity of the Jejunum

In the gizzard and in the ileum, on day 14, the pH was significantly lower in the LC 0.5 dietary group compared to the C and LC 0.8 groups. On day 24, LC supplementation caused a significantly lower pH in the duodenum and a significantly higher pH in the jejunum (LC 0.8). The reason for the pH changes could be the differences in the digesta retention time in the proventriculus–gizzard and the effect of LC on water absorption from the jejunum and ileum. Previous research works demonstrated that the inclusion of moderate amounts of fiber in low-fiber diets might improve chick performance at early ages by reducing gizzard pH and improving the utilization of nutrients [16,42]. Others have reported that insoluble fiber (IF) induced a decrease in proventricular and gizzard pH [38,43]. Many works have also proved that increased cholecystokinin (CCK) production decreased the pH in the proventriculus, and increased bile reflux from the small intestine into the gizzard can affect pepsinogen secretion [43,44,45].

The trypsin activity in the jejunum was not affected by the treatments. The lipase and α-amylase activities at both sampling time points were numerically higher in the LC 0.5 dietary group compared to the C and LC 0.8 groups. The difference was significant only in the case of α-amylase on day 24.

Most of the experiments studying the impact of IF on digestive enzymes have analyzed the effect on pancreatic or intestinal lipases, α-amylases, and peptidases [2,17,43,46]. Recently, the work of Hetland et al. [2,36] corroborated that adding IF in the form of oat hulls and wood shavings to broiler and layer diets improves nutrient digestion possibly as a result of an increased amylase concentration in the chyme of the jejunum [2,36]. Svihus [43] concluded that increased amylase activity and bile acid concentration may also be the reason for the improved nutritive value of diets associated with structural components. Boguslawska-Tryk [21] and Yokhana et al. [16] have demonstrated that increased levels of cellulose in chicken diets increased the proteolytic activity of pancreatic trypsin and chymotrypsin. So, the swelling property of amorphous LC and its effect on the gizzard can stimulate the digestive enzyme secretion of the pancreas.

Although the present work cannot confirm all of these results, the observed significant differences in gizzard and jejunum pH on day 14 and the increased α-amylase activity on day 24 showed that LC supplementation can have similar effects as structural fibers. The reason for the moderate differences in the measured pH and enzyme activities was that the inclusion of LC was low in this trial and cannot be compared with the structural fiber effects of oat hulls, sunflower meal, or wheat bran.

### 4.4. Microbial Diversity and Microbial Abundances of the Ileal Content

The microbiota of the gut is related to the ability of humans and animals to degrade complex carbohydrates. In chickens, the bacterial fermentation of IF sources and lignified materials such as lignocellulose is low [4,9,12,13,14,37].

In this trial, LC did not cause differences in the bacterial alpha diversity of the ileal content. The beta diversity of bacterial communities also showed significant differences only between the two age categories. No such published diversity results were found in the literature. The results are not surprising, since lignocelluloses are assumed not to be degraded in the ileum. LC could modify the bacteriota composition in the small intestine; however, since the control diet contained only highly digestible components, LC probably could not create further improvements in this trial. The differences between the two age categories are well known and correspond to the results of Such et al. [47], who found that the diversity of ileum chymus microbiota in broiler chickens increases with age and reaches its maximum on day 21.

In some previous trials, LC was found to produce significant changes in the bacteriota composition of the caeca. Feeding cellulose products increased the lactic acid-producing bacteria (LAB) and *Bifidobacterium* spp. counts and decreased the abundance of *Escherichia*, *Shigella Escherichia coli*, and *Clostridium perfringens* ratio [18,21,23,39]. The new generation sequencing results of De Maeschalk et al. [48] proved that feeding amorphous cellulose increases the cecal abundance of the phylum *Bacteriodetes* and the genus of *Alistipes*. The authors concluded that cellulose is not entirely inert but can also be a substrate in the chicken’s caeca. Only Makivic et al. [18] investigated the effects of LC on the ileal bacteriota composition of 42-day-old Cobb 500 broiler chickens. Similarly to the aforementioned cecal results, in the ileum, LC also increased the total LAB and *Bifidobacteria*, and it decreased the counts of *Escherichia coli* and *Clostridium perfringens*. The results are not entirely comparable with ours, since culturing was used in that trial and the chickens were different in breed and age. No new generation sequencing results have been found in the literature. In our experiment, the bacteriota composition of ileum was not affected by the LC treatments at the phylum or genus level. Similarly to the diversity results, the bacteriota composition at the genus level showed many significant differences between the two age categories.

## 5. Conclusions

The results of this study showed that feeding LC at low, practical inclusion rates does not modify the weight gain of broiler chickens; however, it can decrease the feed intake and improve the feed-conversion ratio. Interestingly, the chickens responded only in the grower phase, and the 0.5% inclusion rate was more effective in the case of several parameters (gizzard pH and α-amylase activity on day 14) than the 0.8% ratio. The efficiency of the production (EPEF) was also the highest for the 0.5% treatment. Feeding chickens LC products with a high swelling ability increased the retention time in the gizzard, which proved, in this experiment, the significantly lower pH of gizzard content (day 14) and duodenum digesta (day 24) and the differences in the water content of the crop, gizzard, jejunum, and ileum contents. The increased DM of jejunal and ileal digesta on day 14 means that amorphous celluloses modify not only the transit time of the feed but also increase the absorption of water from the small intestine. Except for α-amylase, on day 24, at low inclusion rates, LC did not modify the digestive enzyme secretion of the pancreas and the bacteriota composition of the ileum content. In line with other results, the 0.5% inclusion of Arbocel seems to be the optimal for broiler chickens in the starter and grower phases.

## Figures and Tables

**Table 1:** Composition and measured nutrient content of the experimental diets (g/kg).

	Starter Diets			Grower Diets			Finisher Diets		
Ingredient	C	LC 0.5	LC 0.8	C	LC 0.5	LC 0.8	C	LC 0.5	LC 0.8
Corn	391.2	386.2	383.2	437.2	432.2	429.2	493.3	488.3	485.3
Wheat	100	100	100	100	100	100	100	100	100
Extracted soybean meal	400	400	400	354	354	354	302	302	302
Sunflower oil	55	55	55	65	65	65	63	63	63
Limestone	18	18	18	15	15	15	15	15	15
MCP	16	16	16	14	14	14	13	13	13
L-lysine	4	4	4	2	2	2	2	2	2
DL-methionine	4	4	4	3	3	3	3	3	3
L-threonine	1	1	1	1	1	1	1	1	1
L-valine	1	1	1						
Premix ^1^	5	5	5	4	4	4	3.5	3.5	3.5
Salt	3	3	3	3	3	3	3	3	3
Sodium bicarbonate	1	1	1	1	1	1	1	1	1
Lignocellulose ^2^		5	8		5	8		5	8
Coccidiostat (Maxiban)	0.63	0.63	0.63						
Coccidiostat (Elancoban)				0.55	0.55	0.55			
Phytase ^3^	0.1	0.1	0.1	0.1	0.1	0.1	0.1	0.1	0.1
NSP degrading enzyme ^4^	0.1	0.1	0.1	0.1	0.1	0.1	0.1	0.1	0.1
**Measured nutrient content**									
AMEn (MJ/kg) ^5^	12.74	12.61	12.59	12.81	12.60	12.63	12.87	12.8	12.97
Crude protein	221	225	219	210.9	207.9	210.6	184.7	186.6	184.8
Crude fiber	37.9	41.9	42.8	40.8	42.8	44.2	43.8	50.8	51.3
Crude fat	76.2	78.9	79.9	78.6	77.6	75.5	81.6	84.0	86.3
Starch	362.8	346.0	348.7	367.9	365.8	368.2	405.2	389.7	397.8
Calcium	10.4	11.0	11.1	9.8	9.7	10.0	8.8	9.1	9.2
Total phosphorus	7.2	7.5	7.5	7.1	7.2	7.1	6.1	6.1	6.2
Lysine	15.1	15.4	15.1	12.8	12.7	13.0	11.1	11.4	11.3
Methionine	6.5	6.9	6.7	6.0	5.7	5.8	5.5	5.7	5.4
Cystine	3.4	3.4	3.3	3.2	3.3	3.3	2.9	3.1	2.8
Methionine + cystine	9.9	10.3	10.0	9.2	9.0	9.1	8.4	8.7	8.2
Threonine	8.8	9.2	9.0	8.9	8.5	8.8	7.8	7.5	7.5
Valine	10.9	11.1	10.9	9.5	9.5	9.5	8.3	8.2	8.3
Isoleucine	9.3	9.4	9.4	8.7	7.6	8.9	7.5	7.8	7.5
Arginine	14.5	14.6	14.5	13.9	14.1	13.9	12.6	12.2	12.2

C—control; LC 0.5—0.5% lignocellulose supplement; LC 0.8—0.8% lignocellulose supplement. ^1^ The active ingredients in the premix were as follows (per kg of diet): retinyl acetate—5.0 mg; cholecalciferol—145 μg; DL-alpha-tocopherol acetate—100 mg; menadione—5.5 mg; thiamine—4.0 mg; riboflavin—10.0 mg; pyridoxin HCl—6.1 mg; cyanocobalamin—50 μg; nicotinic acid—77.0 mg; pantothenic acid—24 mg; folic acid—2.7 mg; biotin—240 μg; betaine—250 mg; choline chloride—450 mg; antioxidant—100 mg; Zn (as ZnSO_4_·H_2_O)—110 mg; Cu (as CuSO_4_·5H_2_O)—15 mg; Fe (as FeSO_4_·H_2_O)—75 mg; Mn (as MnO)—110 mg; I (as KI)—2.0 mg; Se (as Na_2_SeO_3_)—400 μg. ^2^ Lignocellulose product: Arbocel^®^ (Rettenmaier & Söhne GmbH + Co. KG, Rosenberg, Germany). ^3^ Phytase: Quantum Blue^®^ 5G (AB Vista, Marlborough, UK). ^4^ NSP enzyme: Econase^®^ XT 25P (AB Vista, Marlborough, UK). ^5^ AMEn values were calculated according to EU regulation 152/2009.

**Table 2:** Effect of dietary treatments on the body weight and weight gain of broiler chickens.

Dietary Treatments	Feed Intake (g/Bird)				Weight Gain (g/Bird)			
	Starter	Grower	Finisher	Total	Starter	Grower	Finisher	Total
C	268	1277 ^A^	2650	4194	230	930	1728	2887
LC 0.5	268	1239 ^B^	2573	4081	235	942	1696	2873
LC 0.8	264	1237 ^B^	2681	4182	227	909	1733	2869
Pooled SEM	2.37	13.39	55.74	64.60	3.48	11.73	32.15	39.85
***p***-Value	0.464	0.087	0.391	0.408	0.299	0.152	0.686	0.942
	Feed-Conversion Ratio (g/g)				Mortality (Nb)			
	Starter	Grower	Finisher	Total	Starter	Grower	Finisher	Total
C	1.17	1.37 ^A^	1.53	1.45	2	3	0	5 (2.5%)
LC 0.5	1.14	1.32 ^B^	1.52	1.42	1	1	0	2 (1.0%)
LC 0.8	1.16	1.36 ^A^	1.55	1.46	1	0	3	4 (2.1%)
Pooled SEM	0.02	0.02	0.02	0.01				
***p***-Value	0.551	0.068	0.523	0.146				

C—control; LC 0.5—0.5%; LC 0.8—0.8% lignocellulose supplement; ^A^, ^B^ Averages with different letter marks show tendencies (*p* ≤ 0.1).

**Table 3:** Impact of dietary lignocellulose on the relative weight and digesta content of the digestive-tract parts and on the dry matter (DM) content of the digesta on day 14.

Gut Segments	Dietary Treatments	Relative Weight of Digestive-Tract Parts (%)			DM Content of Digesta (%)			Digesta Contents of Digestive-Tract Parts (g DM)		
		Mean	Pooled SEM	*p*-Value	Mean	Pooled SEM	*p*-Value	Mean	Pooled SEM	*p*-Value
Crop	C	0.39	0.056	0.226	42.6 ^a^	0.84	0.002	3.0 ^A^	0.39	0.083
	LC 0.5	0.49			39.7 ^ab^			3.2 ^A^		
	LC 0.8	0.36			38.0 ^b^			2.0 ^B^		
Gizzard	C	4.11	0.180	0.656	33.8 ^A^	0.59	0.090	4.0	0.17	0.491
	LC 0.5	4.28			32.1 ^B^			3.8		
	LC 0.8	4.36			32.2 ^B^			3.7		
Duodenum	C	1.22 ^B^	0.056	0.085	21.0	0.83	0.927	0.4	0.05	0.160
	LC 0.5	1.39 ^A^			21.5			0.5		
	LC 0.8	1.36 ^A^			21.3			0.5		
Jejunum	C	2.90	0.083	0.397	17.8 ^b^	0.24	0.000	1.8	0.07	0.120
	LC 0.5	3.07			19.4 ^a^			2.0		
	LC 0.8	2.94			18.2 ^b^			1.9		
Ileum	C	1.73	0.051	0.973	19.4 ^B^	0.20	0.073	2.1	0.07	0.174
	LC 0.5	1.70			19.8 ^AB^			2.2		
	LC 0.8	1.71			20.1 ^A^			2.3		

C—control; LC 0.5—0.5%; LC 0.8—0.8% lignocellulose supplement. The production parameters were evaluated with one-way analysis of variance (ANOVA). ^a^, ^b^ Means with different superscripts of the same column are significantly different. The differences were considered significant at a level of *p* ≤ 0.05. ^A^, ^B^ Averages with different letter marks show tendencies (*p* ≤ 0.1).

**Table 4:** Impact of dietary lignocellulose on the relative weight and digesta content of digestive-tract parts and on the dry matter (DM) content of the digesta on day 24.

Gut Segments	Dietary Treatment	Weight of Digestive-Tract Parts (g)			DM Content of the Gut Contents (%)			Digesta Contents of Digestive-Tract Parts (g DM)		
		Mean	Pooled SEM	*p*-Value	Mean	Pooled SEM	*p*-Value	Mean	Pooled SEM	*p*-Value
Crop	C	0.54	0.066	0.454	37.9	0.85	0.196	2.1	0.57	0.580
	LC 0.5	0.64			38.0			1.3		
	LC 0.8	0.57			35.7			1.4		
Gizzard	C	2.22	0.118	0.201	33.5 ^a^	0.32	0.047	4.5	0.43	0.367
	LC 0.5	2.21			33.3 ^ab^			5.3		
	LC 0.8	2.54			32.4 ^b^			5.1		
Duodenum	C	0.99	0.058	0.612	17.4	0.80	0.877	0.5	0.04	0.393
	LC 0.5	0.91			16.9			0.5		
	LC 0.8	0.91			17.0			0.4		
Jejunum	C	1.71	0.053	0.237	20.2 ^a^	0.44	0.002	3.0	0.15	0.430
	LC 0.5	1.60			18.1 ^b^			2.7		
	LC 0.8	1.78			18.3 ^b^			2.8		
Ileum	C	1.29	0.052	0.832	20.0 ^A^	0.28	0.069	3.0	0.20	0.739
	LC 0.5	1.26			19.1 ^B^			2.8		
	LC 0.8	1.27			19.7 ^AB^			3.0		

C—control; LC 0.5—0.5%; LC 0.8—0.8% lignocellulose supplement. The production parameters were evaluated with one-way analysis of variance (ANOVA). ^a^, ^b^ Means with different superscripts of the same column are significantly different. The differences were considered significant at a level of *p* ≤ 0.05. ^A^, ^B^ Averages with different letter marks show tendencies (*p* ≤ 0.1).

**Table 5:** Impact of dietary lignocellulose on the dry matter (DM) content of the excreta.

Dietary Treatments	DM Content of Excreta (%)					
	Day 14			Day 24		
	Mean	Pooled Sem	*p*-Value	Mean	Pooled SEM	*p*-Value
C	18.8	0.228	0.279	16.7	0.347	0.931
LC 0.5	17.8			16.9		
LC 0.8	18.2			16.6		

**Table 6:** Digesta pH of the different digestive-tract parts.

Gut Segments	Dietary Treatment	Digesta pH on Day 14			Digesta pH on Day 24		
		Mean	Pooled SEM	*p*-Value	Mean	Pooled SEM	*p*-Value
Crop	C	5.97	0.08	0.237	5.36	0.06	0.617
	LC 0.5	5.83			5.43		
	LC 0.8	5.77			5.38		
Gizzard	C	3.69 ^a^	0.09	0.011	3.75	0.10	0.524
	LC 0.5	3.35 ^b^			3.79		
	LC 0.8	3.70 ^a^			3.90		
Duodenum	C	6.37 ^b^	0.02	0.045	6.31 ^a^	0.02	0.011
	LC 0.5	6.44 ^ab^			6.24 ^b^		
	LC 0.8	6.45 ^a^			6.27 ^b^		
Jejunum	C	6.30	0.03	0.249	6.25 ^b^	0.03	0.010
	LC 0.5	6.23			6.32 ^ab^		
	LC 0.8	6.30			6.38 ^a^		
Ileum	C	7.17 ^a^	0.07	0.027	7.11	0.11	0.936
	LC 0.5	6.90 ^b^			7.16		
	LC 0.8	7.02 ^a^			7.13		

C—control; LC 0.5—0.5%; LC 0.8—0.8% lignocellulose supplement. The production parameters were evaluated with one-way analysis of variance (ANOVA). ^a^, ^b^ Means with different superscripts of the same column are significantly different. The differences were considered significant at a level of *p* ≤ 0.05.

**Table 7:** Digestive enzyme activity of the jejunum.

Dietary Treatments	Day 14			Day 24		
	Trypsin Units/mg Protein	Lipase Units/mg Protein	α-Amylase Units/mg Protein	Trypsin Units/mg Protein	Lipase Units/mg Protein	α-Amylase Units/mg Protein
C	89.50	0.20	8.18	81.52	0.17	5.81 ^ab^
LC 0.5	82.87	0.21	9.07	76.74	0.19	7.90 ^a^
LC 0.8	89.51	0.20	8.65	79.51	0.17	5.14 ^b^
Pooled SEM	5.760	0.018	0.681	7.447	0.014	0.716
***p***-Value	0.649	0.784	0.659	0.902	0.411	0.033

C—control; LC 0.5—0.5%; LC 0.8—0.8% lignocellulose supplement. α-Amylase: One unit will liberate 1.0 mg of maltose from starch in 3 min at pH 6.9 at 20 °C. Lipase: One unit will hydrolyze 1.0 micro equivalent of fatty acid from a triglyceride in one hour at pH 7.7 at 37 °C. (This is equivalent to approximately 10 microliters of CO_2_ in 30 min.). Trypsin: One BAEE unit will produce a ∆A253 nm of 0.001 per minute with BAEE as substrate at pH 7.6 at 25EC in a reaction volume of 3.2 mL. ^a^, ^b^ Means with different superscripts of the same column are significantly different.

**Table 8:** Relative abundances of bacterial phyla in the ileum.

	Treatments	Age			FDR *p*-Values		
		D14	D24	Mean (Dietary Treatment)	Mean (Dietary Treatment)	Mean (Age)	Interaction
*Actinobacteria*	C	3.60	4.83	4.22	0.977		1.210
	LC 0.5	4.29	4.40	4.34			
	LC 0.8	5.30	5.45	5.38			
	Mean (age)	4.40	4.90			0.705	
*Bacteroidetes*	C	0.00	0.00	0.00	0.788		1.122
	LC 0.5	0.00	0.00	0.00			
	LC 0.8	0.01	0.01	0.01			
	Mean (age)	0.01	0.01			0.808	
*Cyanobacteria*	C	0.11	0.08	0.10	0.708		0.840
	LC 0.5	0.09	0.16	0.12			
	LC 0.8	0.08	0.16	0.12			
	Mean (age)	0.09	0.13			0.243	
*Firmicutes*	C	95.31	94.78	95.04	0.836		0.967
	LC 0.5	95.38	95.08	95.23			
	LC 0.8	94.00	94.10	94.05			
	Mean (age)	94.90	94.65			0.812	
*Patescibacteria*	C	0.10	0.13	0.11	0.914		0.660
	LC 0.5	0.07	0.17	0.12			
	LC 0.8	0.10	0.11	0.10			
	Mean (age)	0.09	0.14			0.182	
*Proteobacteria*	C	0.88	0.17	0.00	0.686		0.740
	LC 0.5	0.17	0.16	0.00			
	LC 0.8	0.51	0.12	0.00			
	Mean (age)	0.52	0.34			0.182	
*Verrucomicrobia*	C	0.00	0.01	0.00	0.917		0.917
	LC 0.5	0.00	0.03	0.02			
	LC 0.8	0.00	0.04	0.02			
	Mean (age)	0.00 ^b^	0.03 ^a^			0.000	

^a^, ^b^ Means with different superscripts of the same row are significantly different. The differences were considered significant at a level of *p* ≤ 0.05.

**Table 9:** Relative abundances of bacterial genera higher than 1% abundance in the ileum.

	Treatments	Age			FDR *p*-Values		
		D14	D24	Mean (Dietary Treatment)	Mean (Dietary Treatment)	Mean (Age)	Interaction
*Lactobacillus*	C	67.44	85.97	76.70	0.701		0.986
	LC 0.5	75.07	85.62	80.34			
	LC 0.8	64.46	81.44	72.95			
	Mean (age)	68.99 ^b^	84.34 ^a^			0.000	
*Staphylococcus*	C	9.65	1.08	5.36	0.950		0.987
	LC 0.5	9.25	0.94	5.09			
	LC 0.8	10.98	1.57	6.28			
	Mean (age)	9.96 ^a^	1.20 ^b^			0.000	
*Streptococcus*	C	5.68	3.54	4.61	0.701		0.986
	LC 0.5	3.35	5.80	4.57			
	LC 0.8	6.03	7.62	6.83			
	Mean (age)	5.02	5.65			0.770	
*Corynebacterium_1*	C	2.56	3.57	3.07	0.886		0.986
	LC 0.5	3.28	3.57	3.43			
	LC 0.8	3.86	3.73	3.80			
	Mean (age)	3.24	3.63			0.710	
*Enterococcus*	C	5.91	0.46	3.19	0.701		0.986
	LC 0.5	3.23	0.31	1.77			
	LC 0.8	6.17	0.38	3.27			
	Mean (age)	5.10 ^a^	0.38 ^b^			0.000	
*Candidatus_* *Arthromitus*	C	2.47	0.10	1.29	0.886		0.986
	LC 0.5	1.59	0.25	0.92			
	LC 0.8	2.49	0.11	1.30			
	Mean (age)	2.18 ^a^	0.15 ^b^			0.000	

^a^, ^b^ Means with different superscripts of the same column are significantly different. The differences were considered significant at a level of *p* ≤ 0.05.

## Data Availability

Raw-sequences data of 16S rRNA gene analysis were deposited at the National Center for Biotechnology Information (NCBI) Sequence Read Archive (SRA) under accession number PRJNA 922062. All data generated or analyzed during this study are included in this published article.
